# Supplementary material for: Glycolysis-dependent sulfur metabolism orchestrates morphological plasticity and virulence in fungi
Source: eLife. 2026 Feb 6;14:RP109075. doi: 10.7554/eLife.109075 (PMC12880806; doi:10.7554/eLife.109075)

Met4-HA/HA

Figure 2E – Met4 levels in SLAD and SLAD+2DG condition

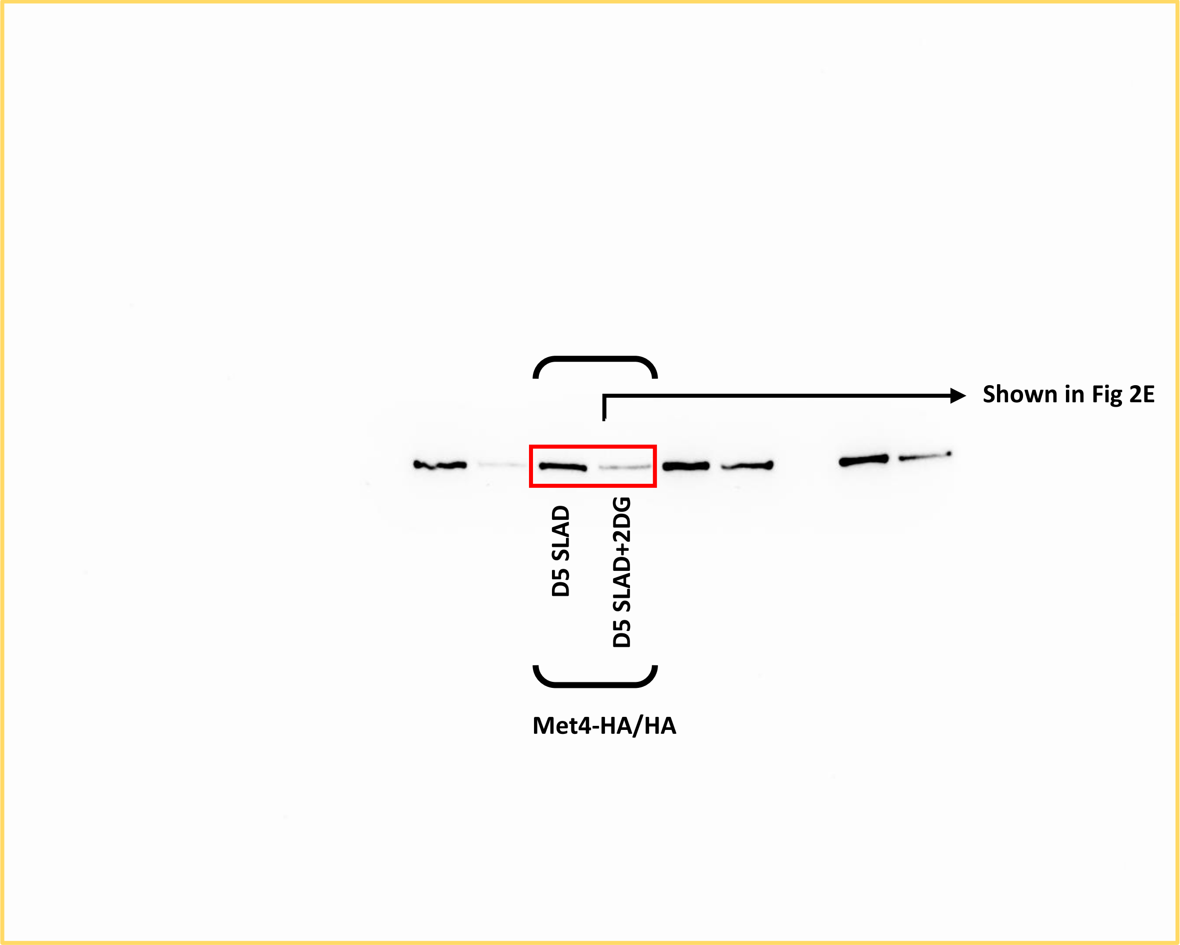

Loading Control-β-actin

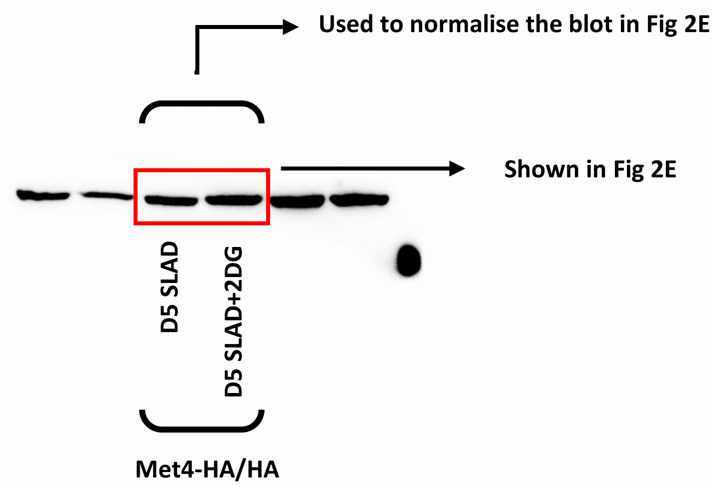

Met32-HA/HA

Figure 2E – Met32 levels in SLAD and SLAD+2DG condition

*

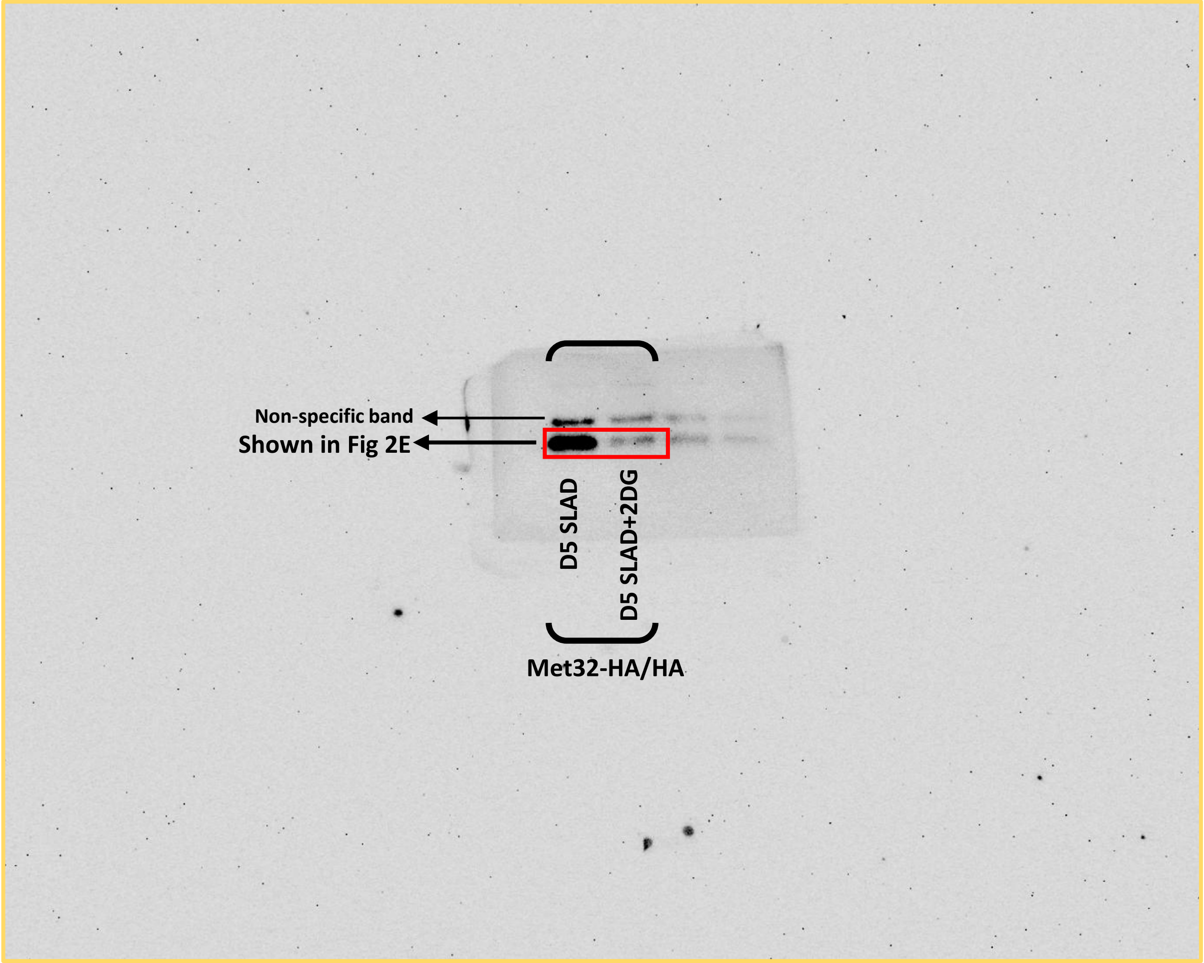

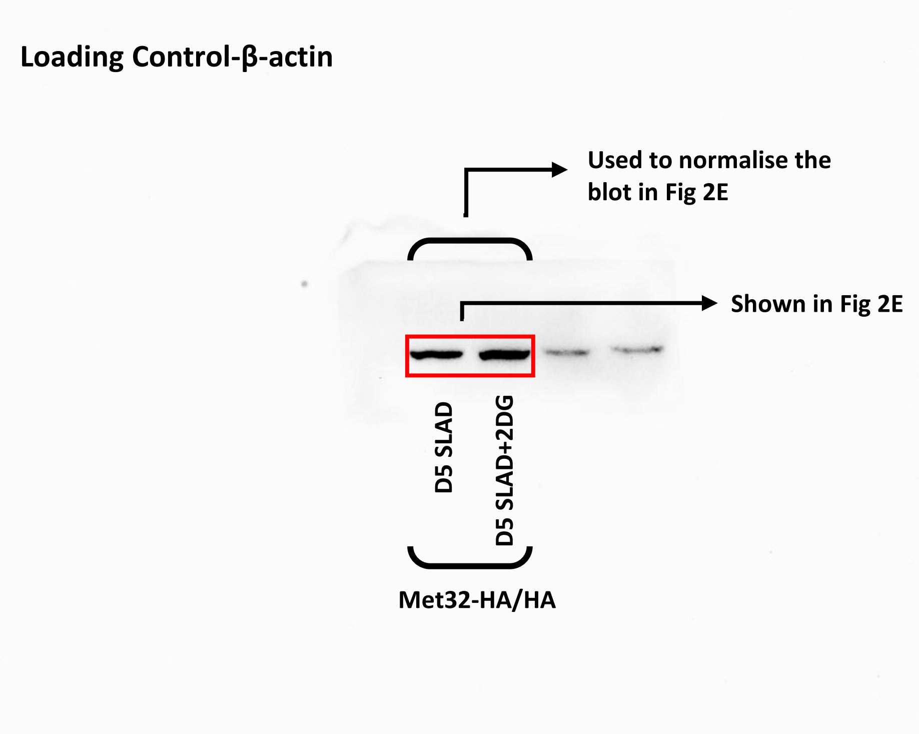

Met16-HA/HA

Figure 2E – Met16 levels in SLAD and SLAD+2DG condition

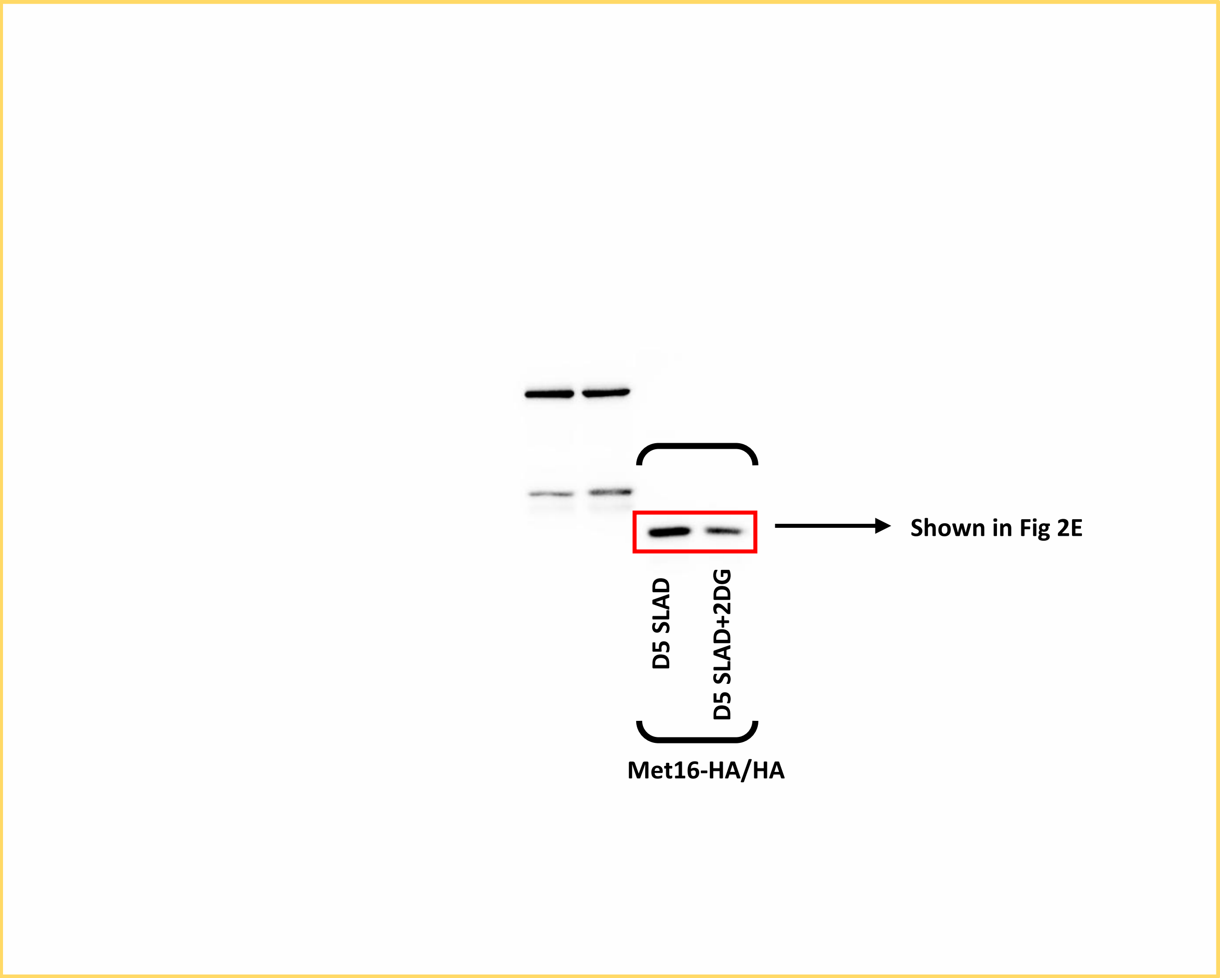

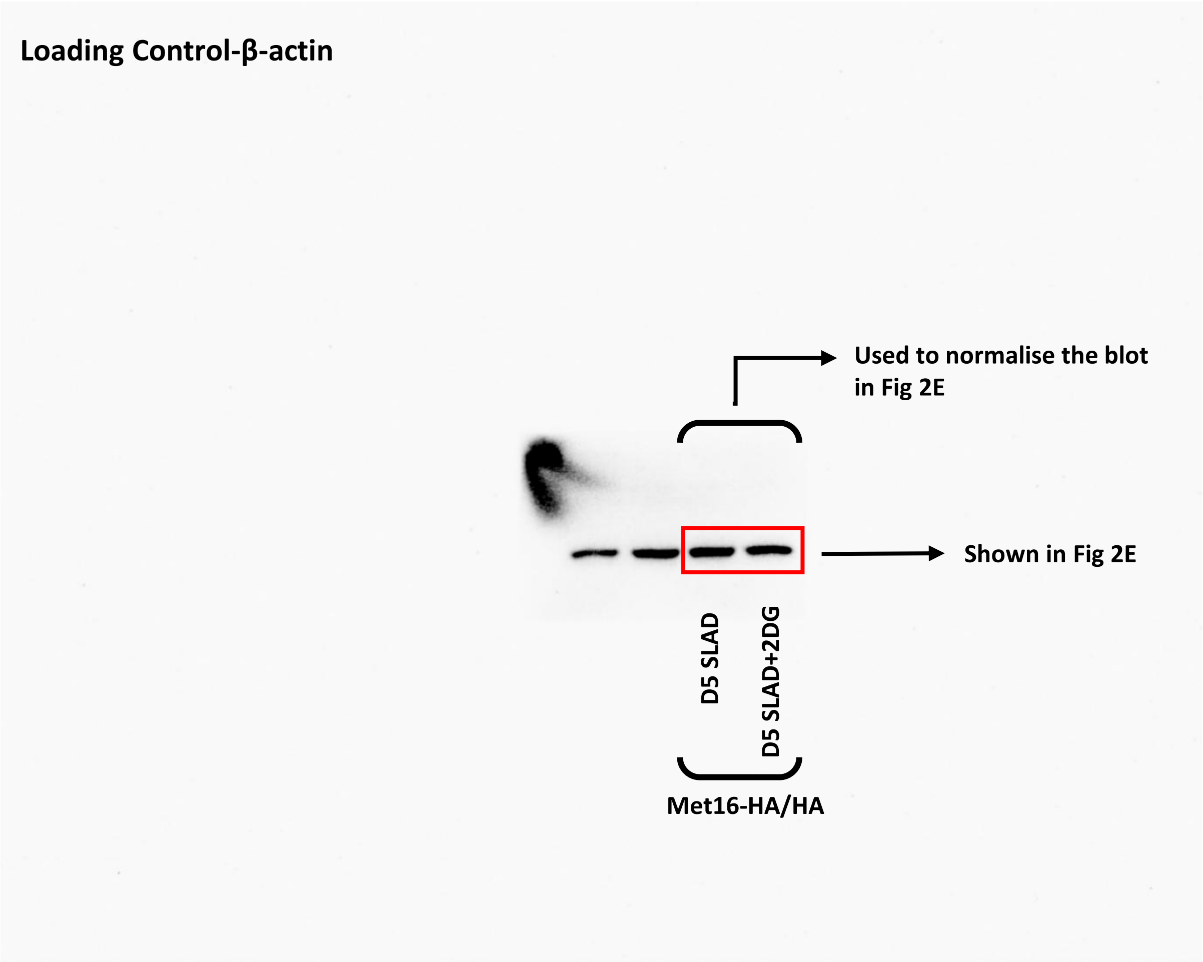

Met10-HA/HA

Figure 2E – Met10 levels in SLAD and SLAD+2DG condition

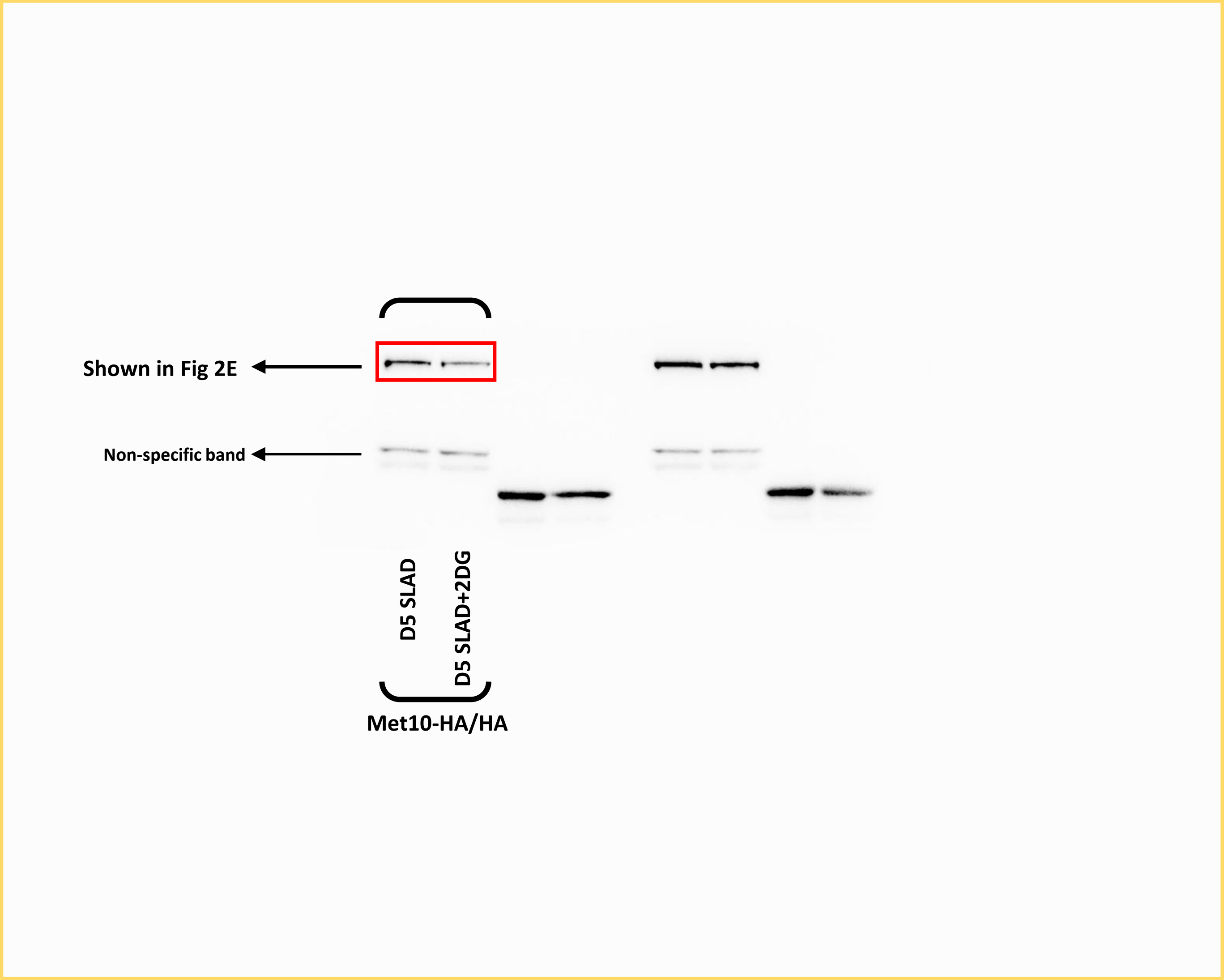

Loading Control-β-actin

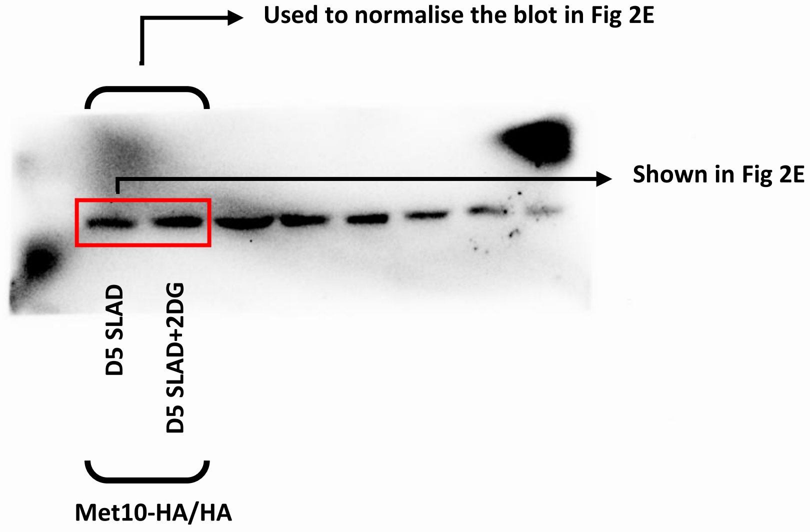

Cys4-HA/HA

Figure 2E – Cys4 levels in SLAD and SLAD+2DG condition

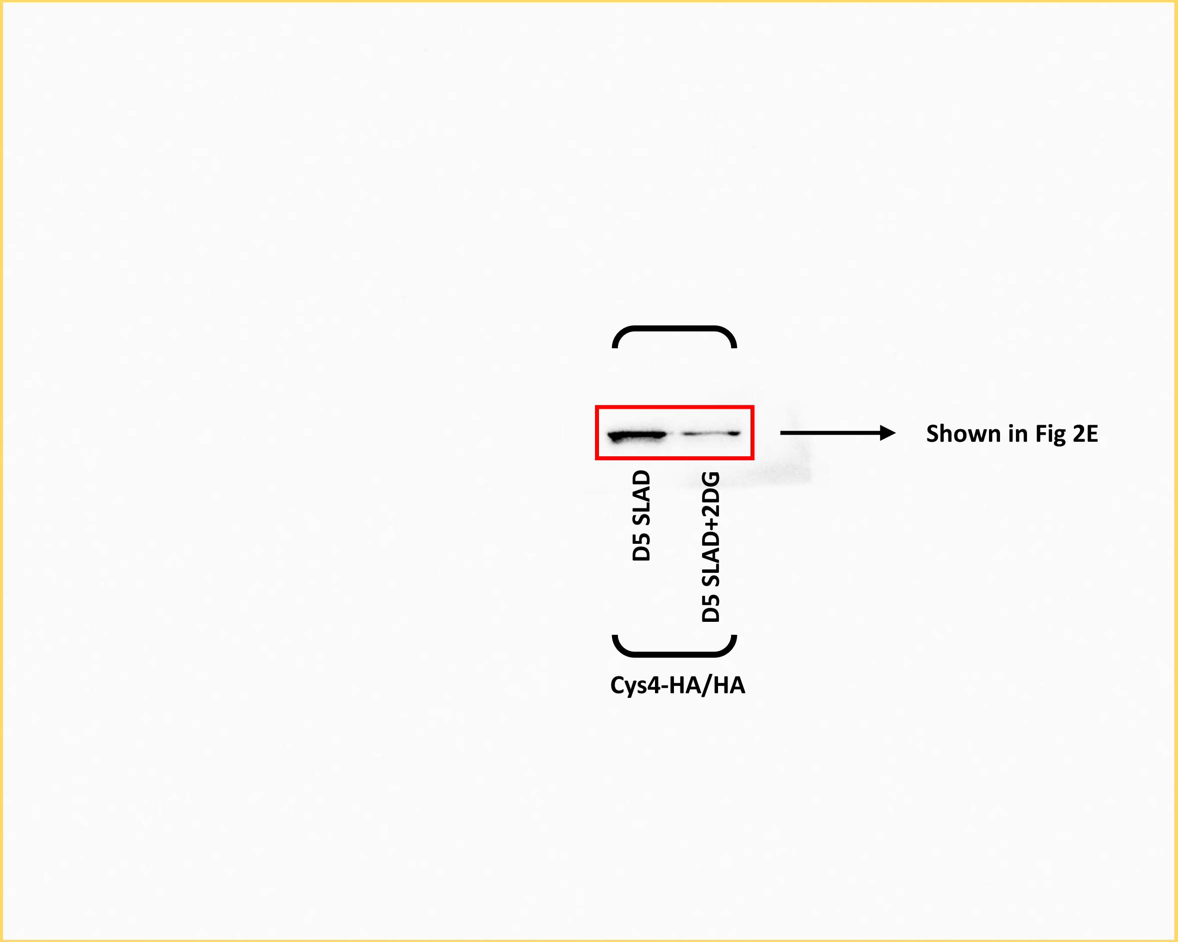

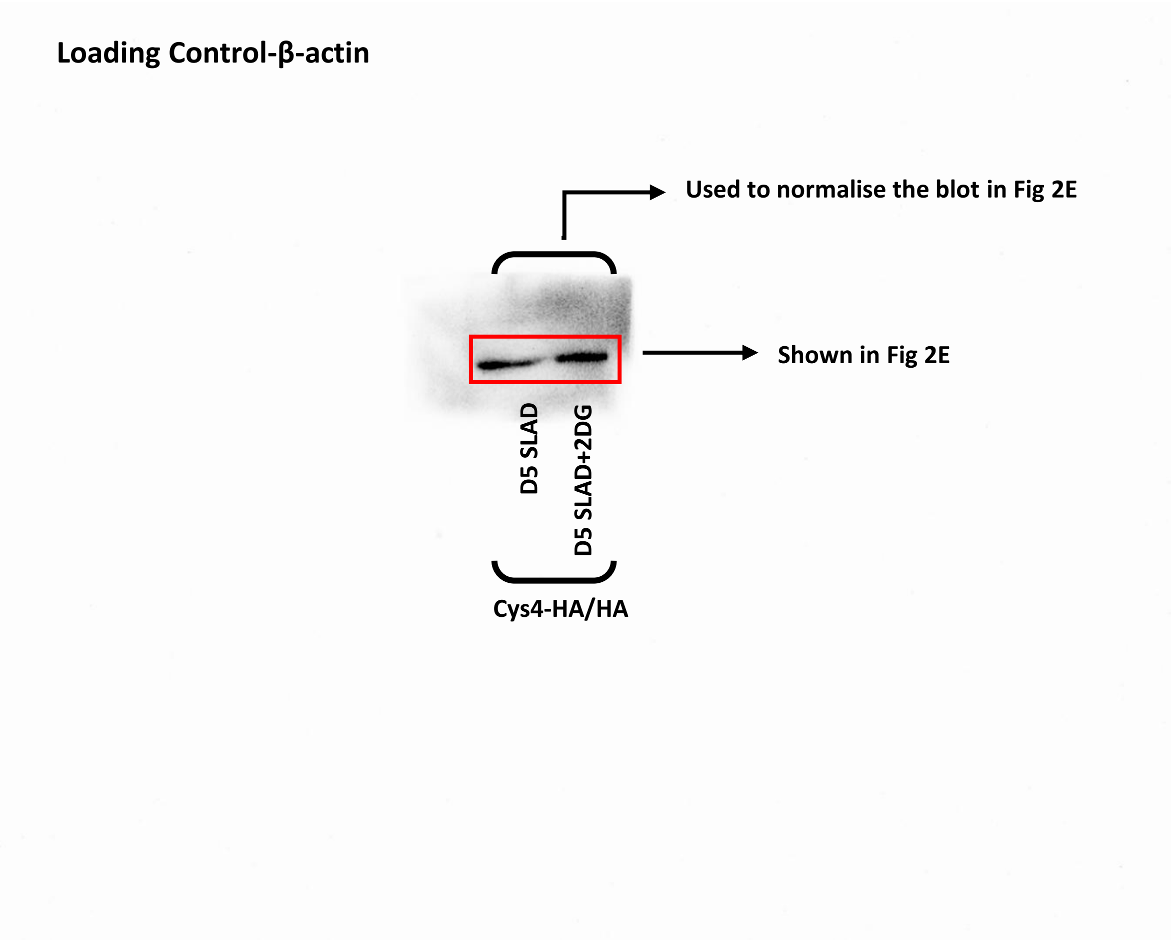

Cys3-HA/HA

Figure 2E – Cys3 levels in SLAD and SLAD+2DG condition

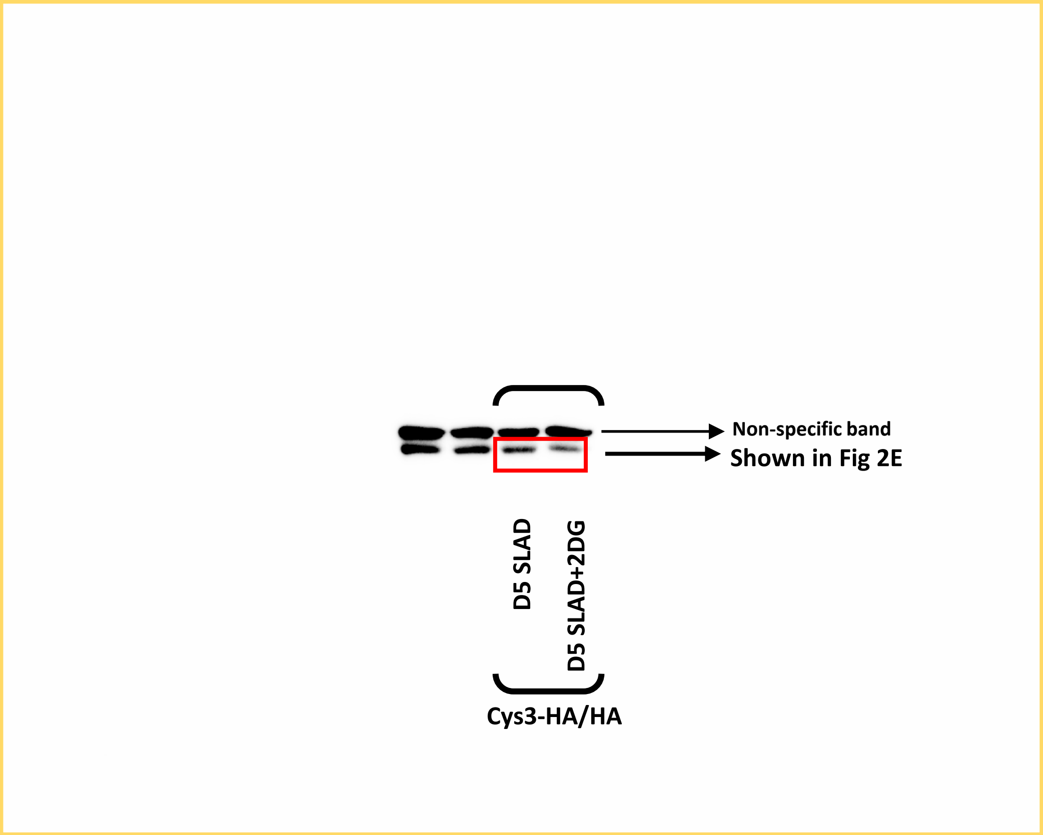

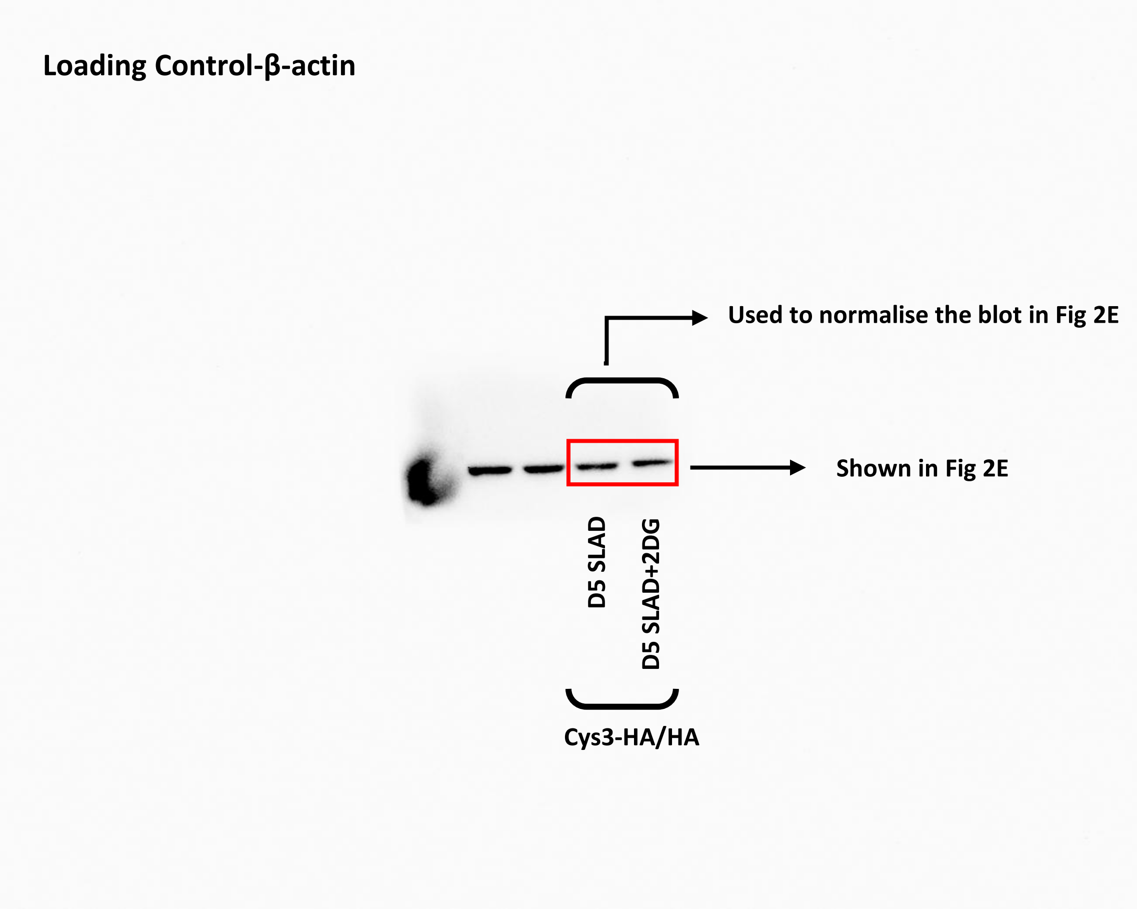

Supplement: Figure 2—source data 1. [file elife-109075-fig2-data1.zip › Figure 2-source Data 1/Figure 2-source data, uncropped and labelled blots.pdf]
