# Supplementary material for: Glycolysis-dependent sulfur metabolism orchestrates morphological plasticity and virulence in fungi
Source: eLife. 2026 Feb 6;14:RP109075. doi: 10.7554/eLife.109075 (PMC12880806; doi:10.7554/eLife.109075)

Met32-HA/HA

Figure 2-figure supplement 2B – Met32 levels in SLAD and SLAD+2DG condition in liquid medium

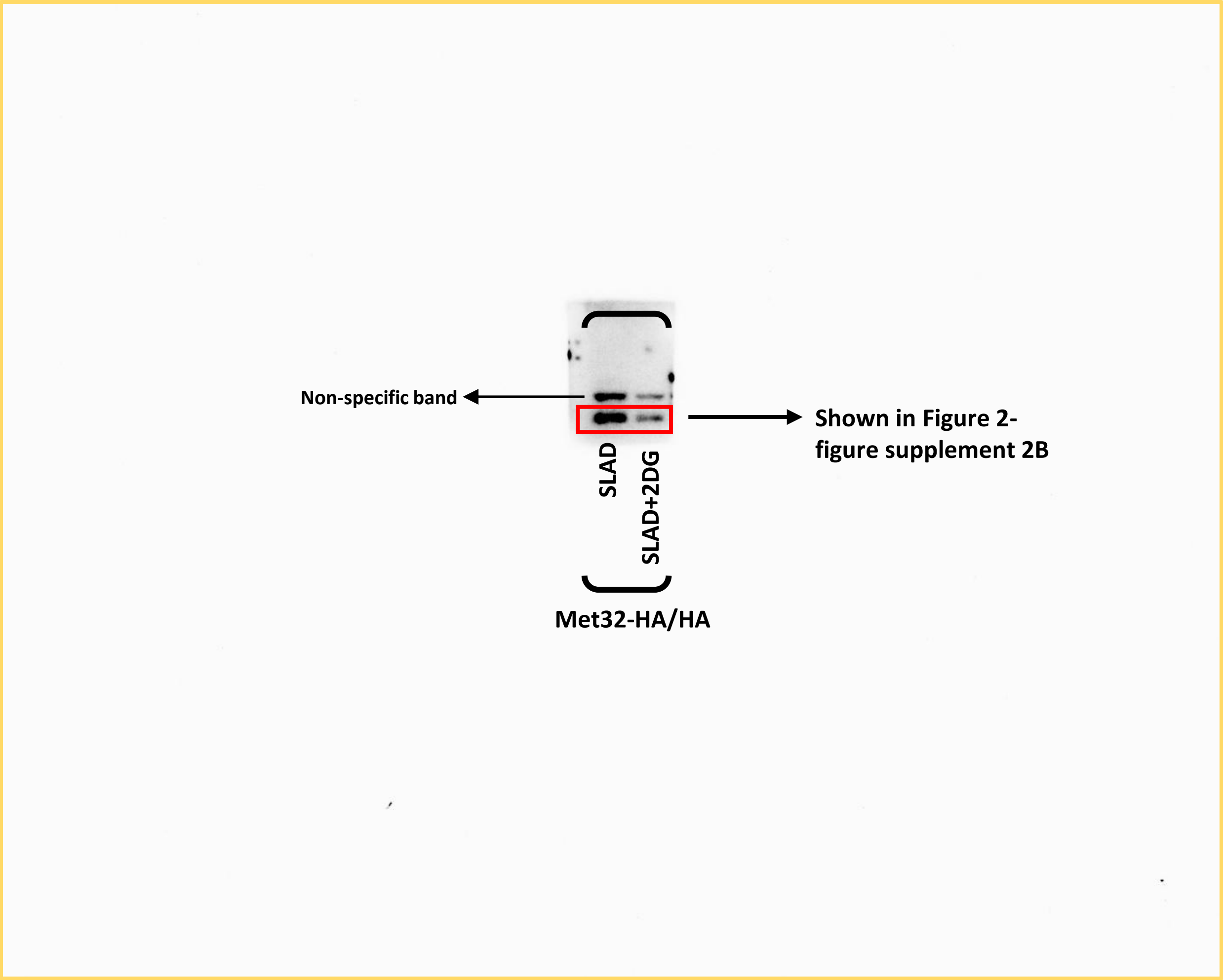

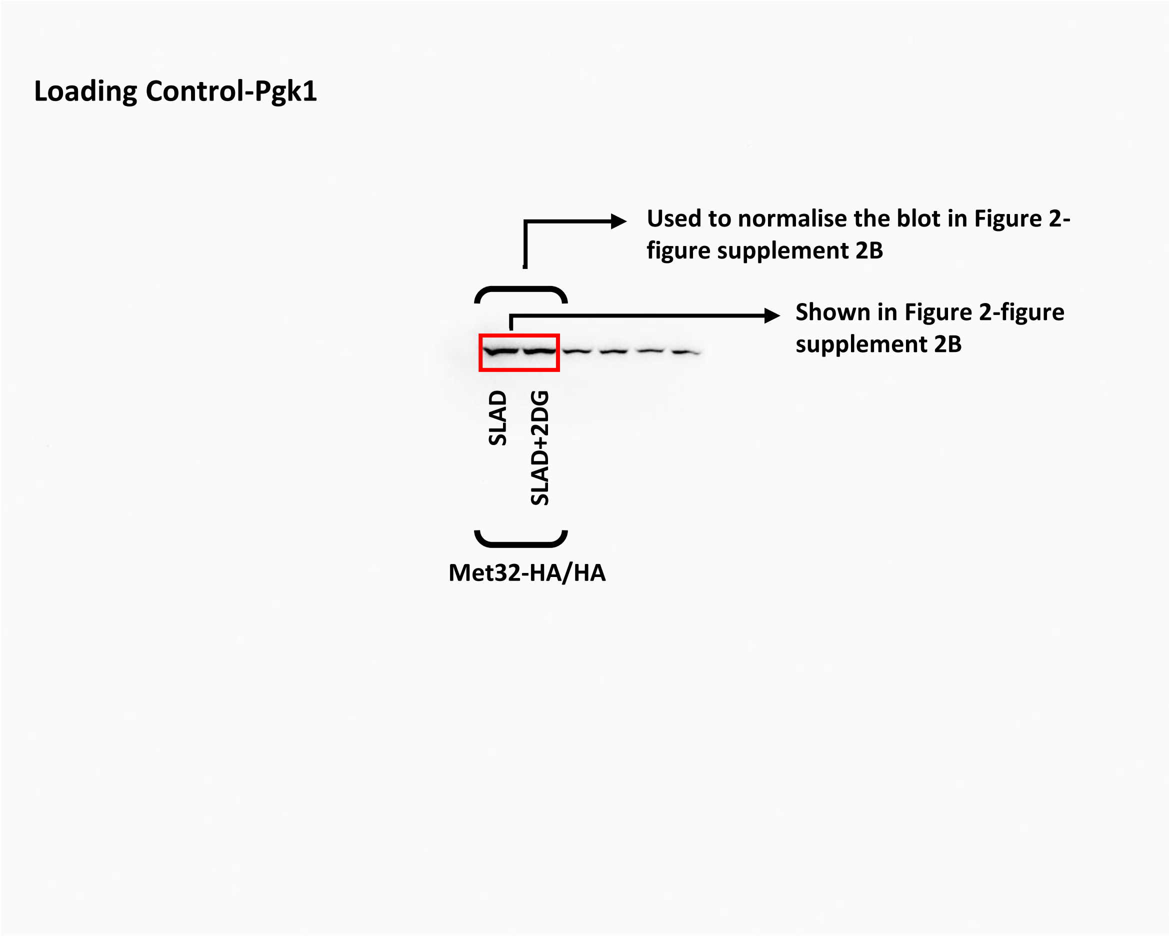

Met16-HA/HA

Figure 2-figure supplement 2B – Met16 levels in SLAD and SLAD+2DG condition in liquid medium

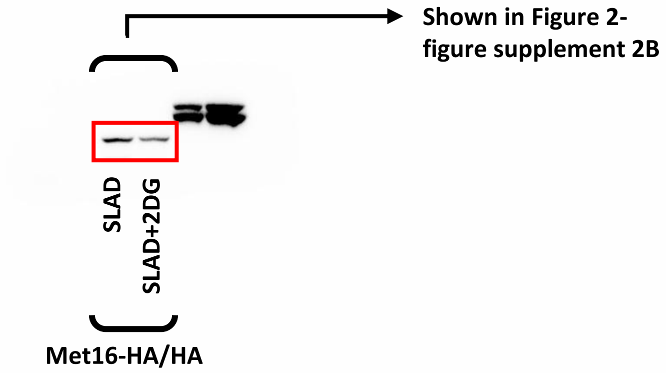

Loading Control-Pgk1

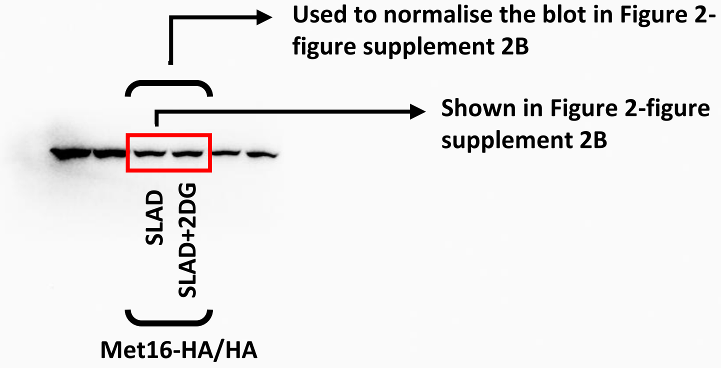

Met10-HA/HA

Figure 2-figure supplement 2B – Met10 levels in SLAD and SLAD+2DG condition in liquid medium

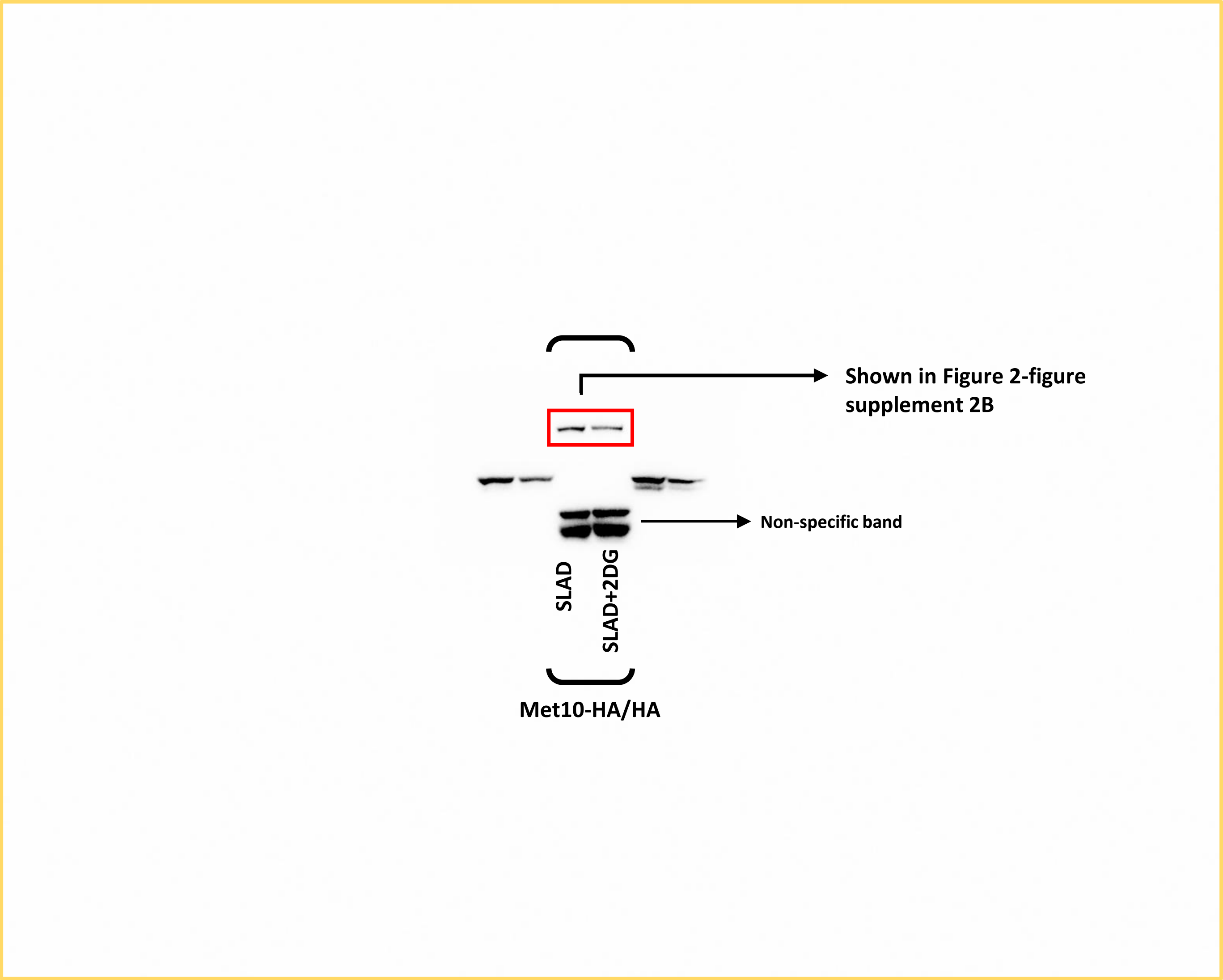

Loading Control-Pgk1

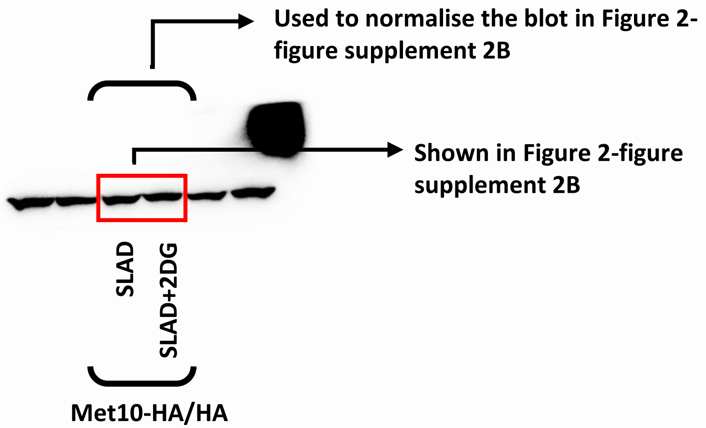

Cys3-HA/HA

Figure 2-figure supplement 2B – Cys3 levels in SLAD and SLAD+2DG condition in liquid medium

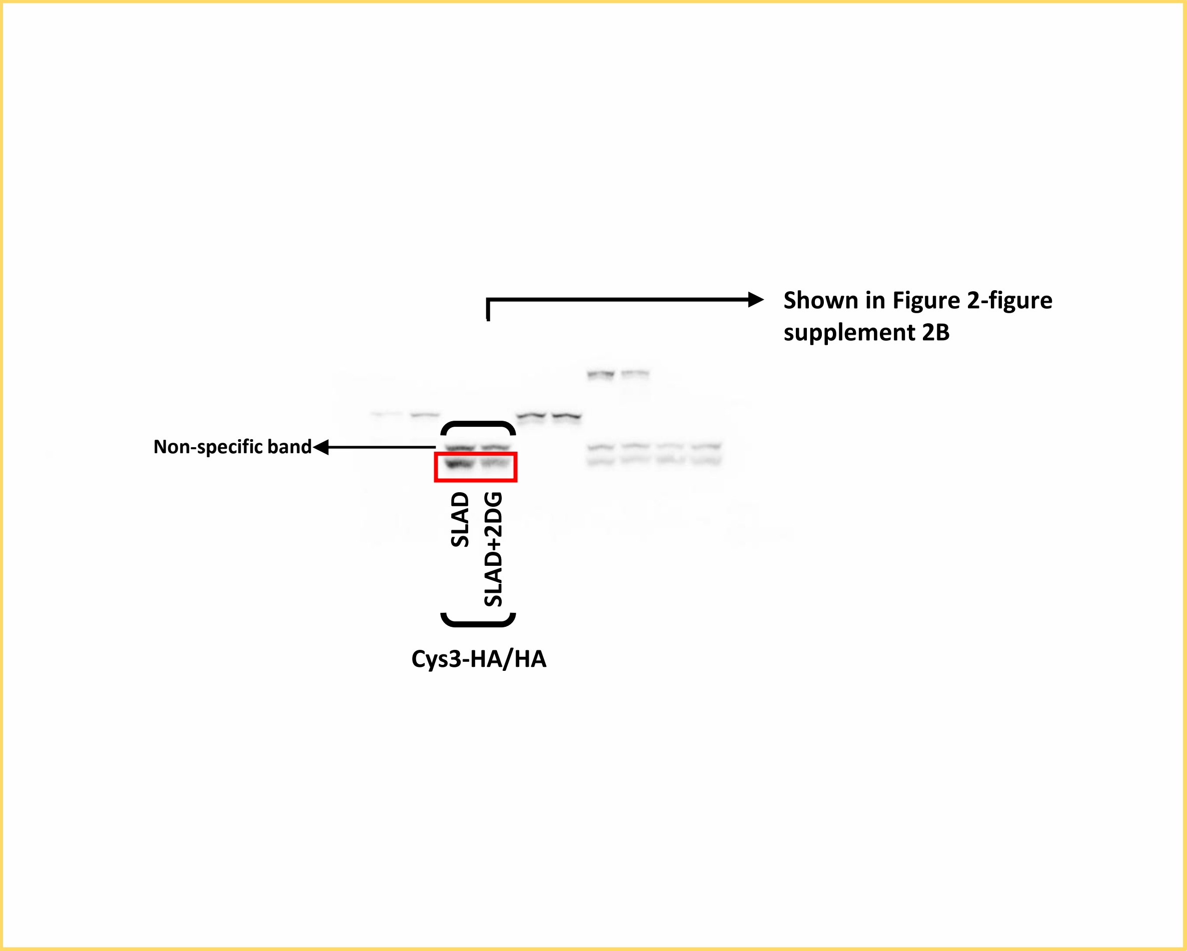

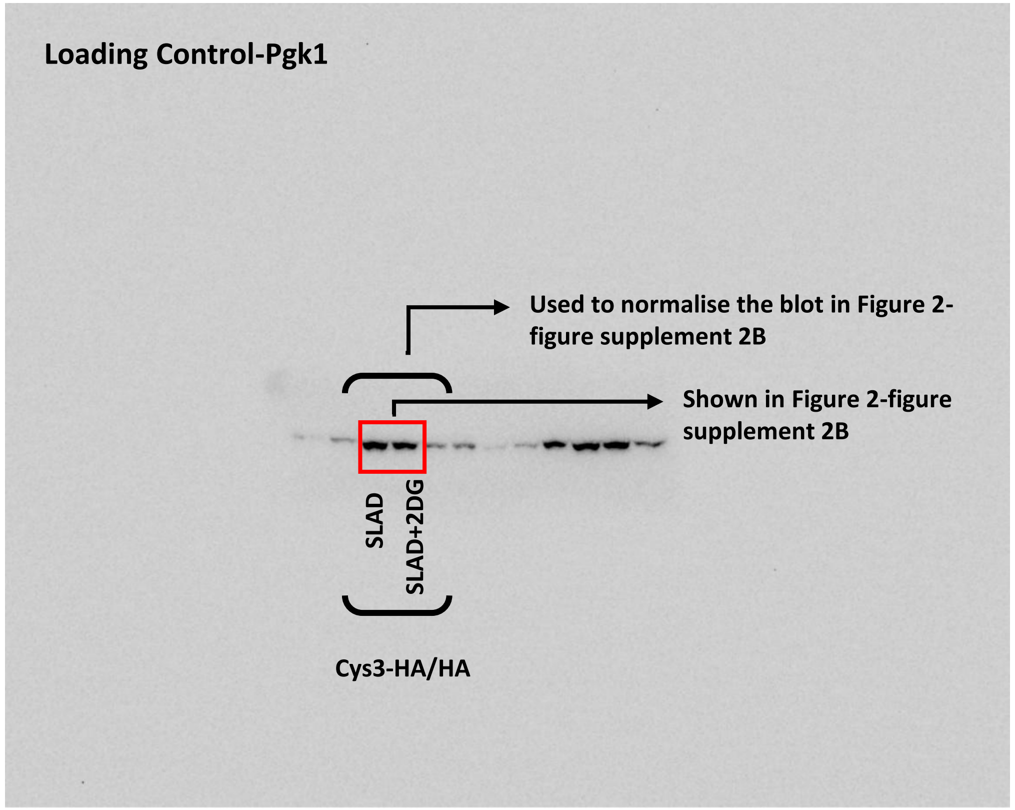

Supplement: Figure 2—figure supplement 2—source data 1. [file elife-109075-fig2-figsupp2-data1.zip › Figure 2-figure supplement 2-source data 1/Figure 2-figure supplement 2-uncropped and labelled blots.pdf]
