# Supplementary material for: Glycolysis-dependent sulfur metabolism orchestrates morphological plasticity and virulence in fungi
Source: eLife. 2026 Feb 6;14:RP109075. doi: 10.7554/eLife.109075 (PMC12880806; doi:10.7554/eLife.109075)

# *Δmet30*/Met4-HA/HA

**Figure 3H – Met4 levels in SLAD and SLAD+2DG condition**

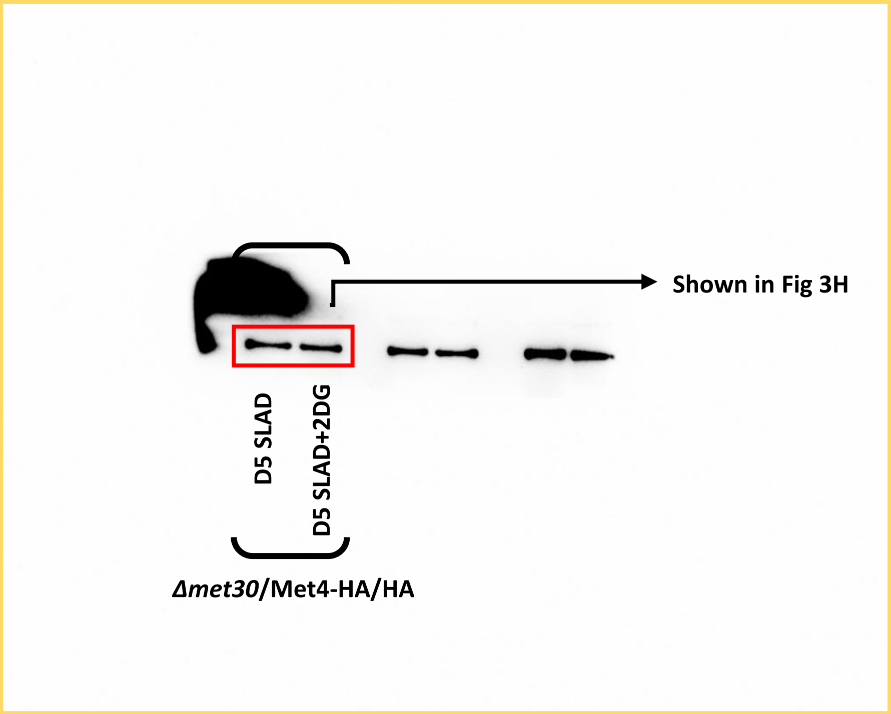


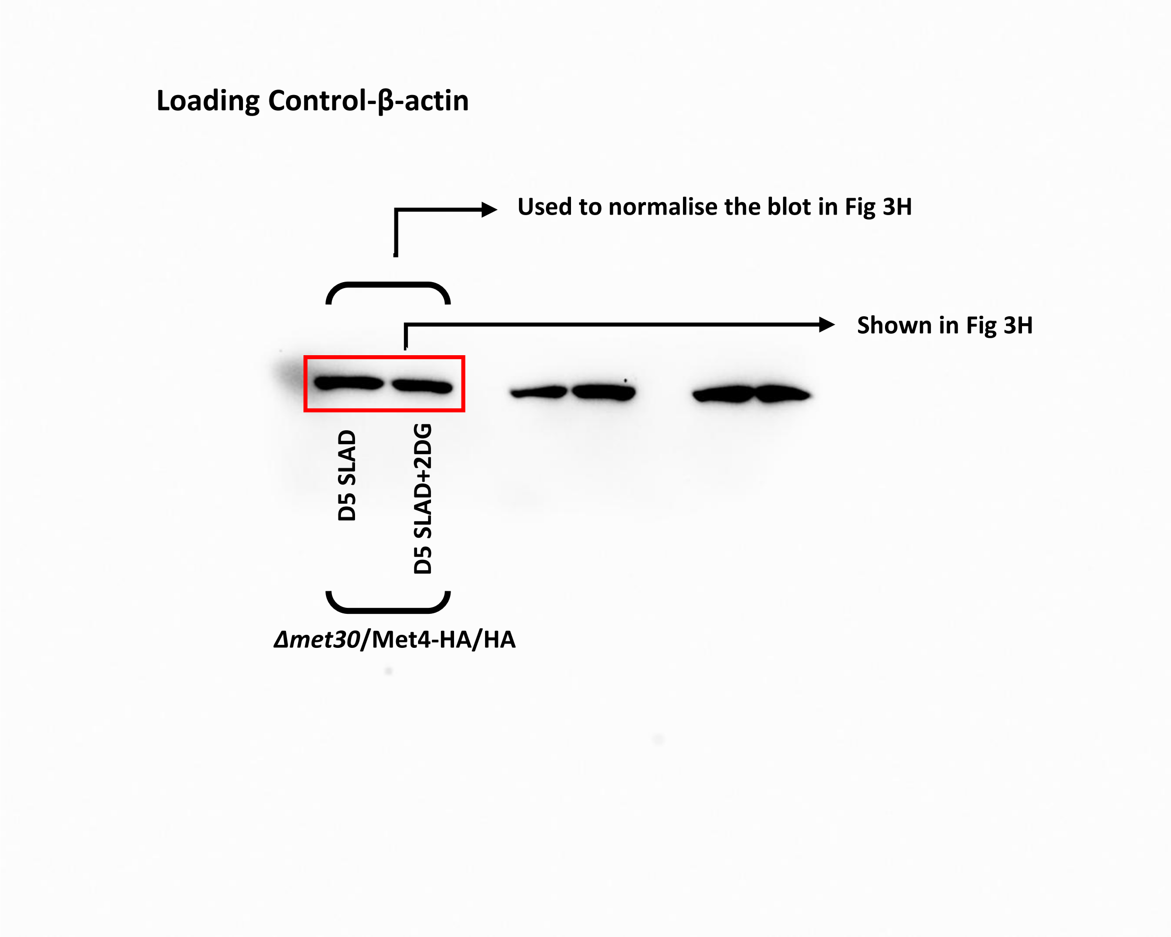

*Δmet30*/Met32-HA/HA

**Figure 3H – Met32 levels in SLAD and SLAD+2DG condition**

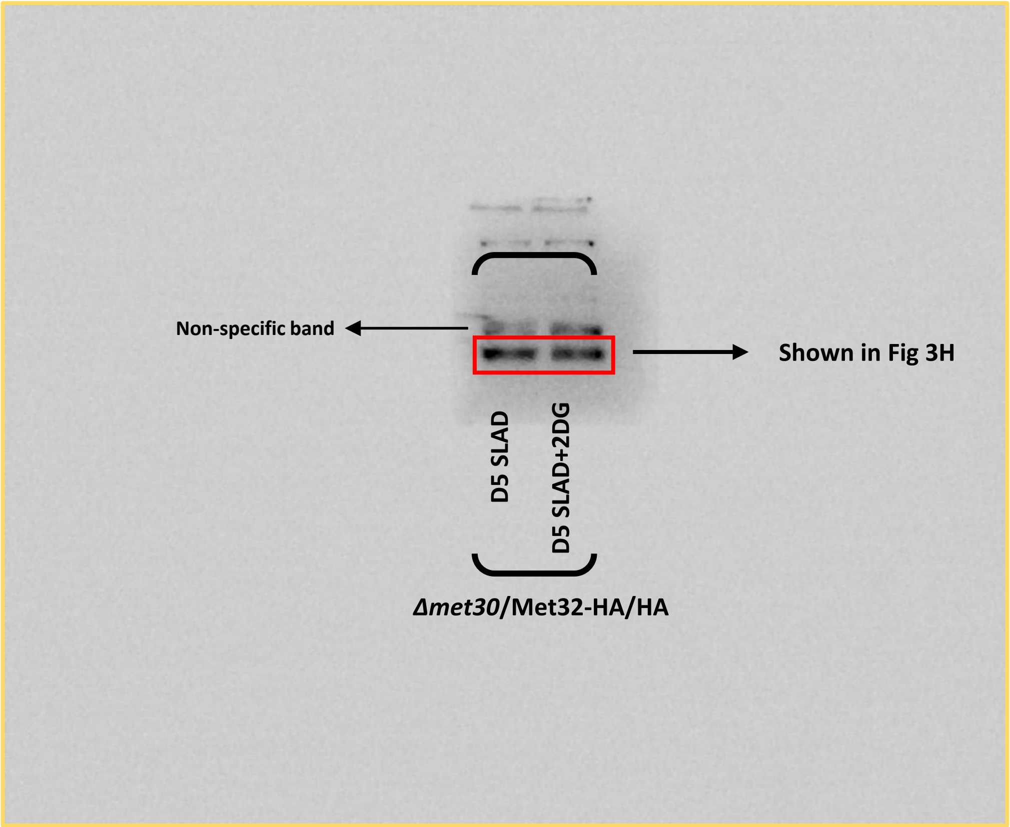


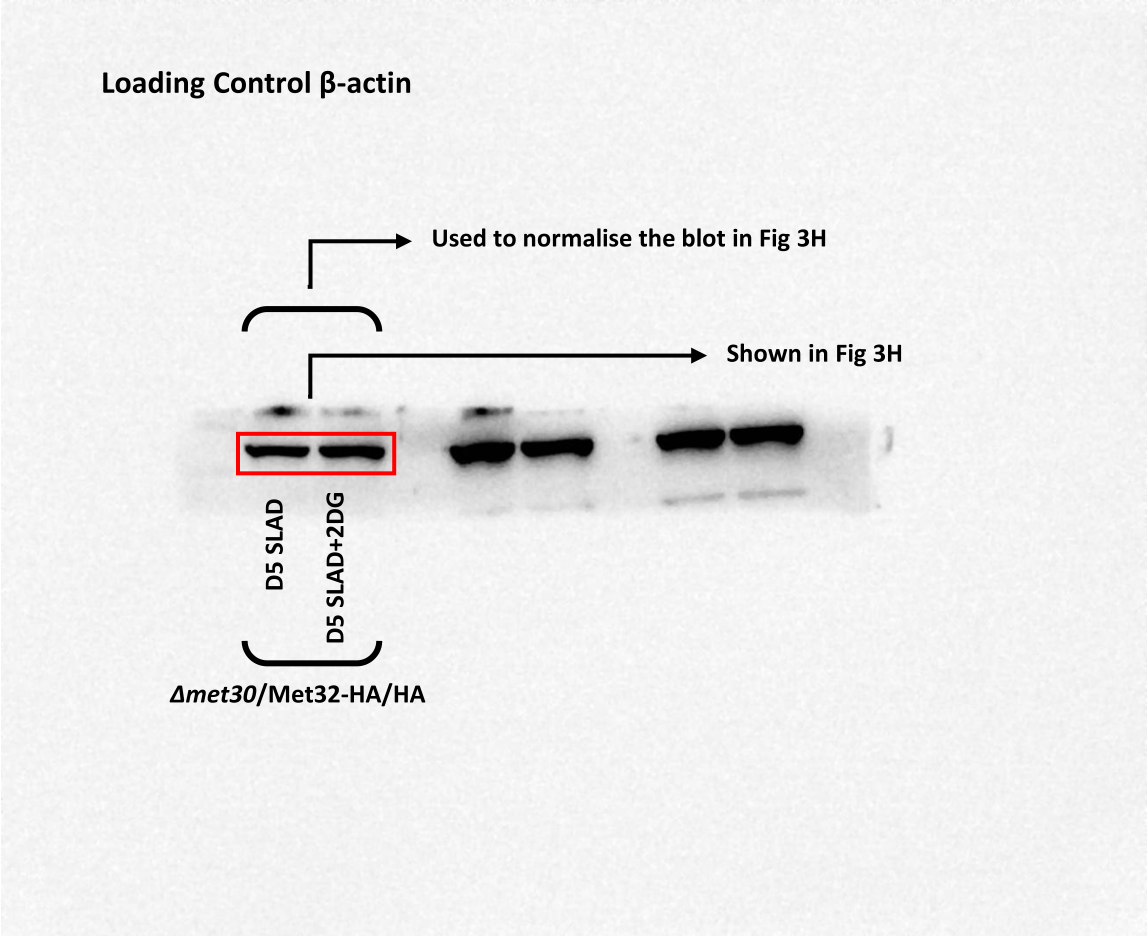

*Δmet30*/Met16-HA/HA

**Figure 3H – Met16 levels in SLAD and SLAD+2DG condition**

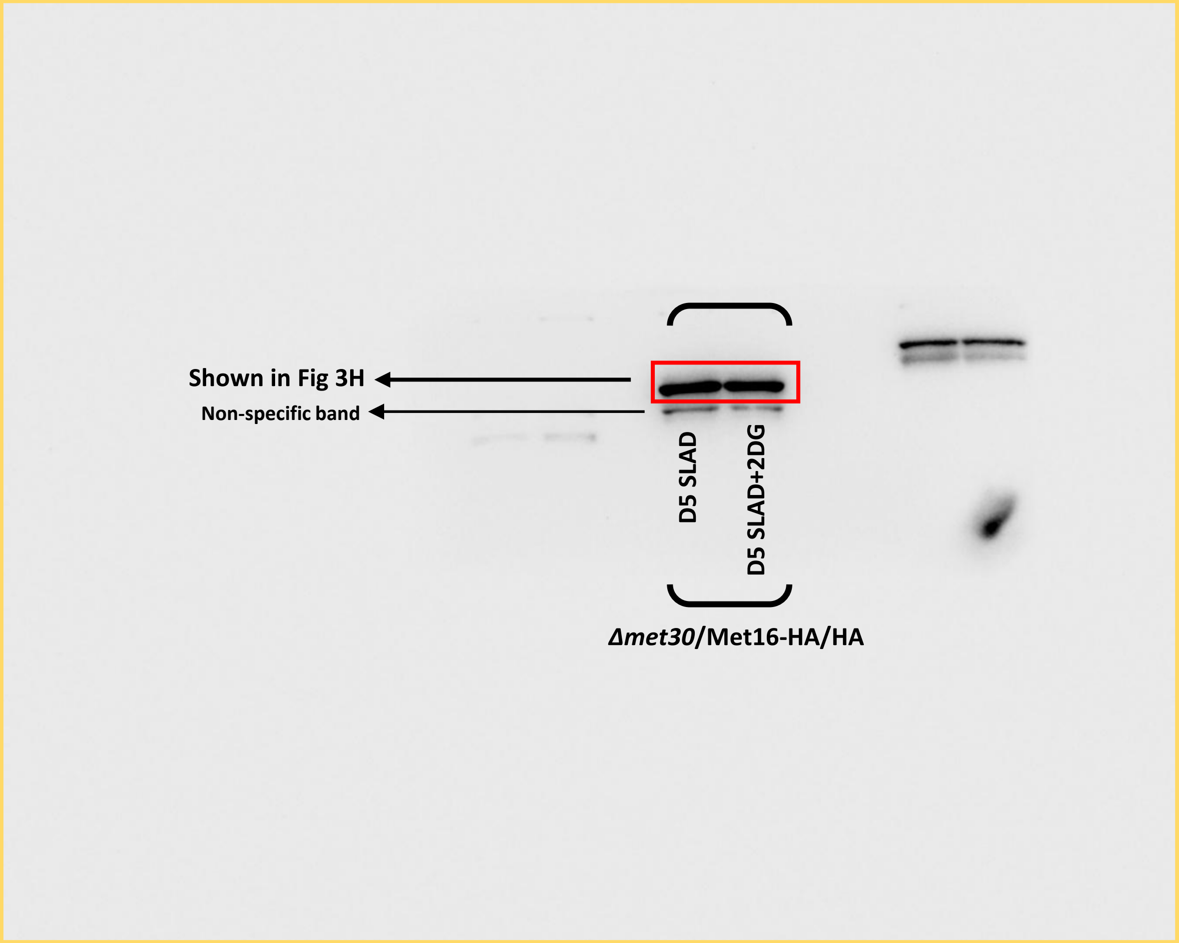


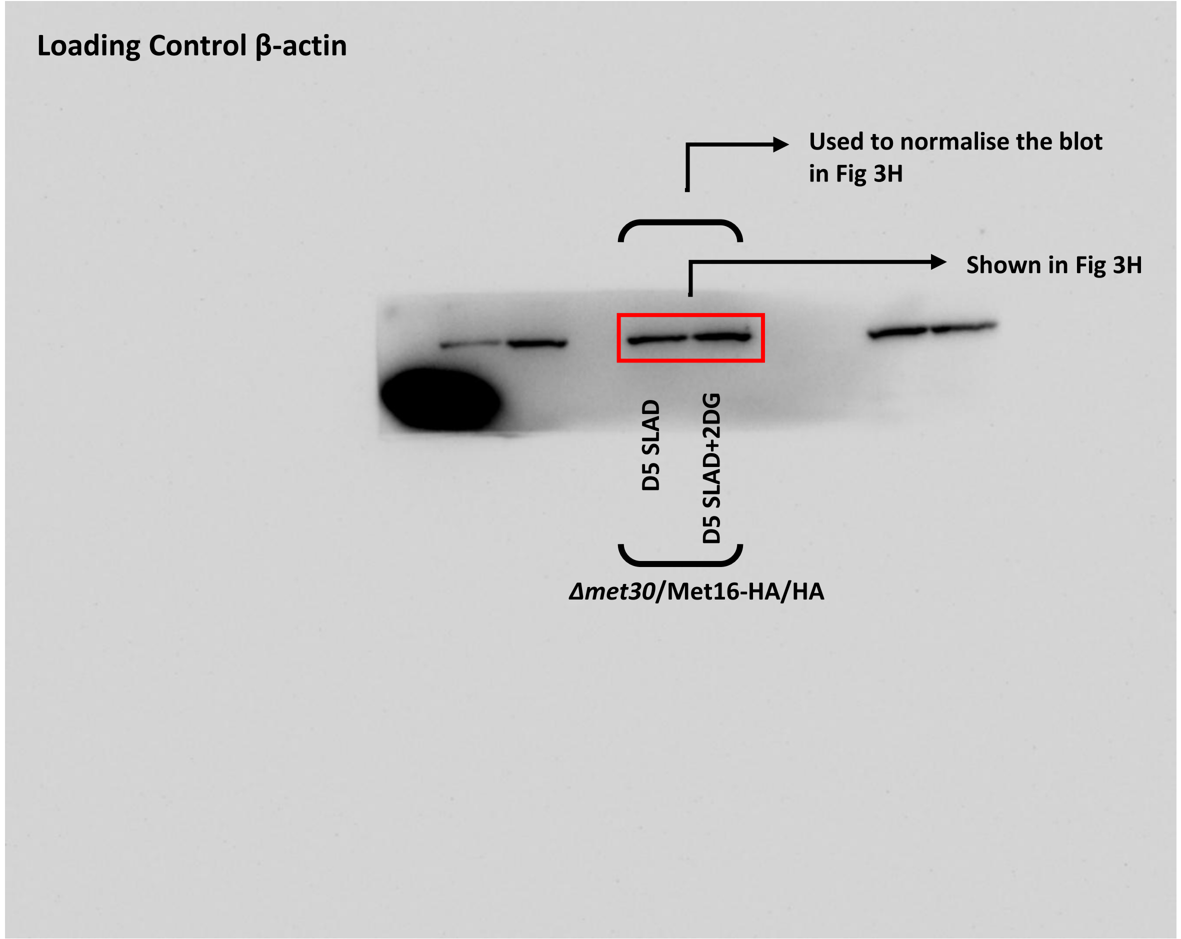

# *Δmet30*/Cys3-HA/HA

**Figure 3H – Cys3 levels in SLAD and SLAD+2DG condition**

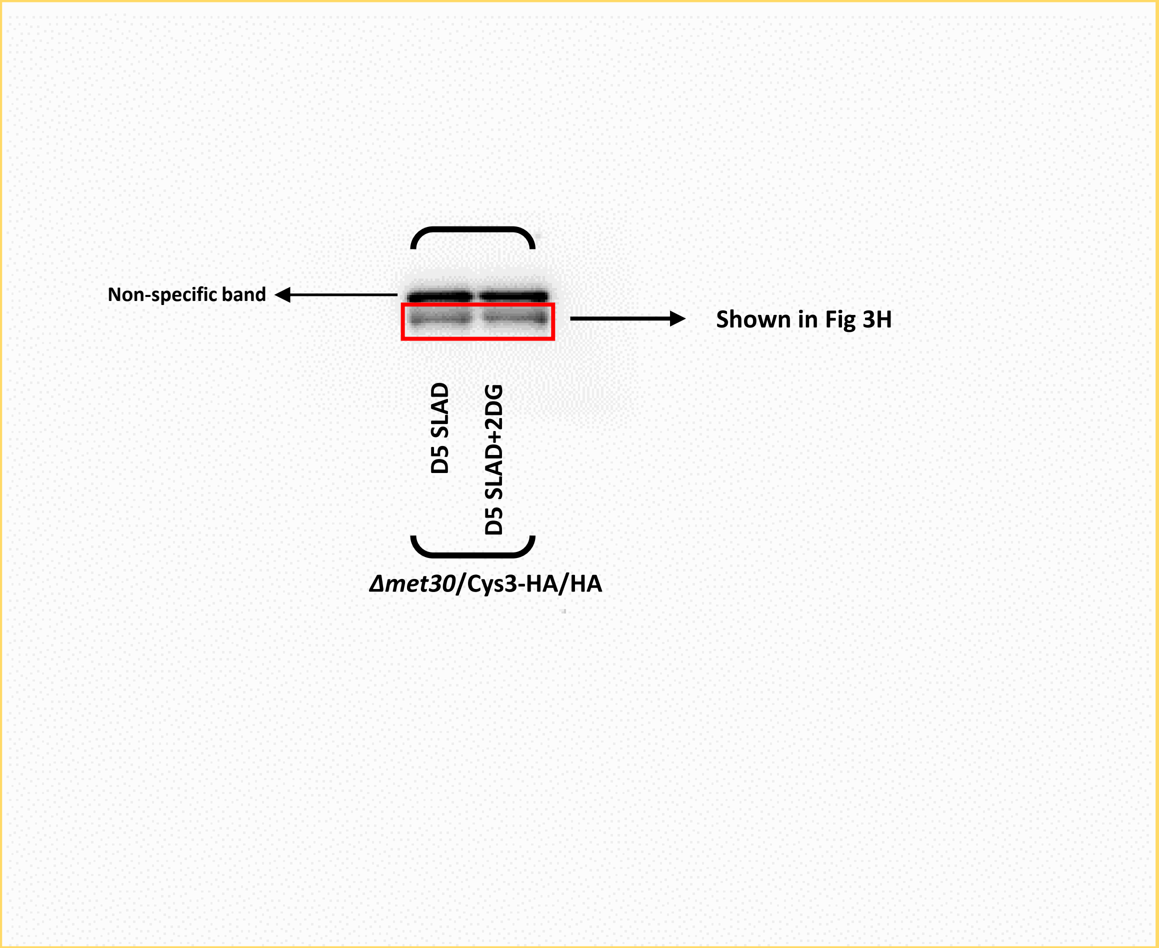


**Loading Control β-actin**

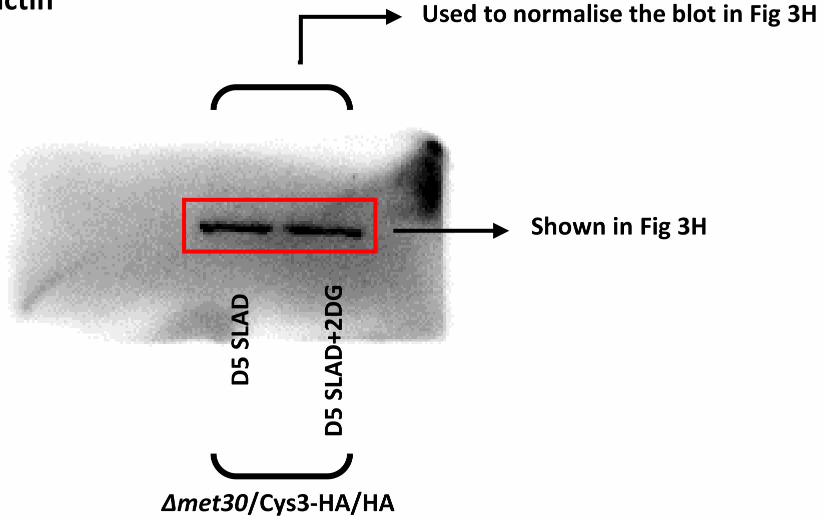

# Met30-HA/HA

**Figure 3J – Met30 levels in SLAD and SLAD+2DG condition**

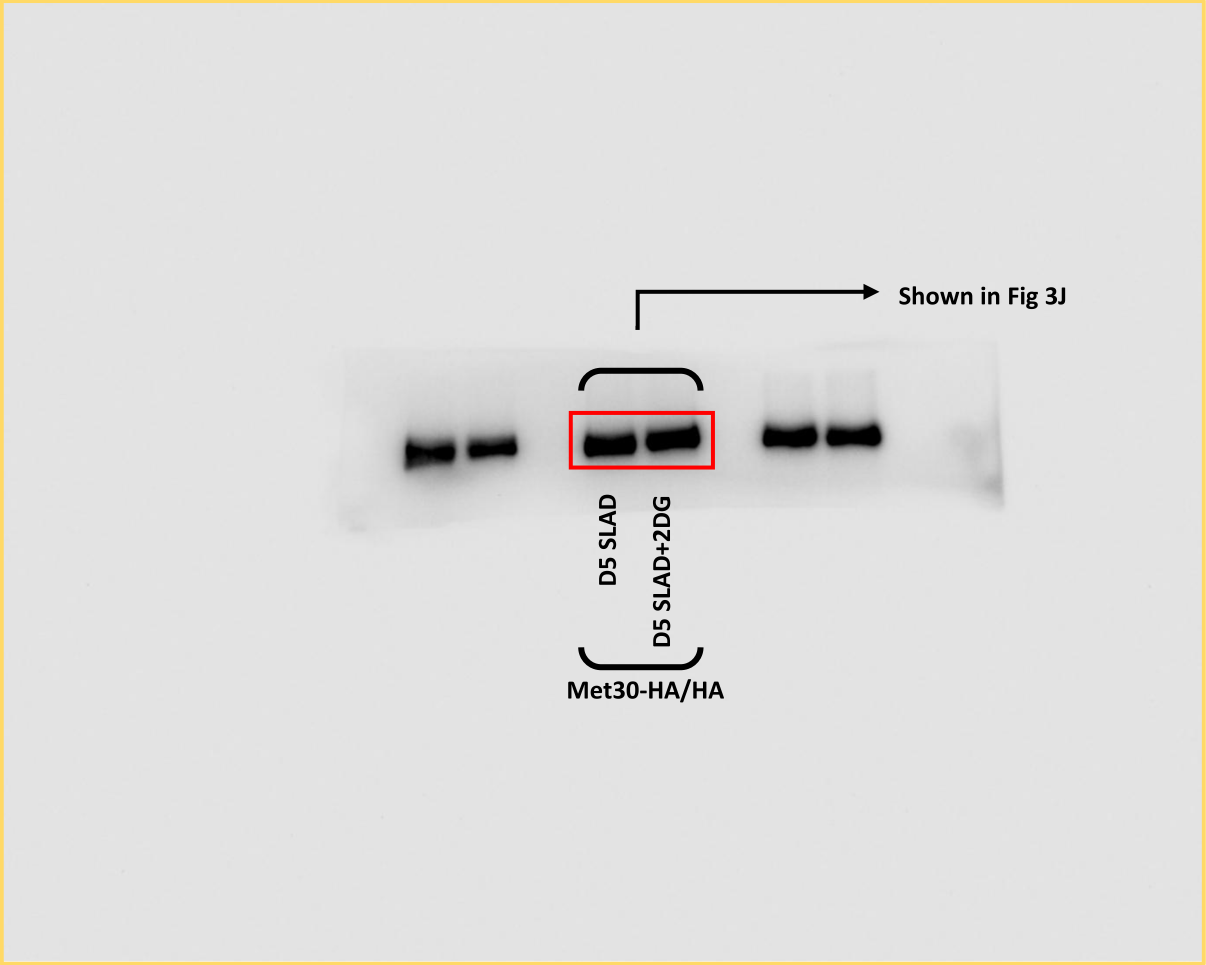


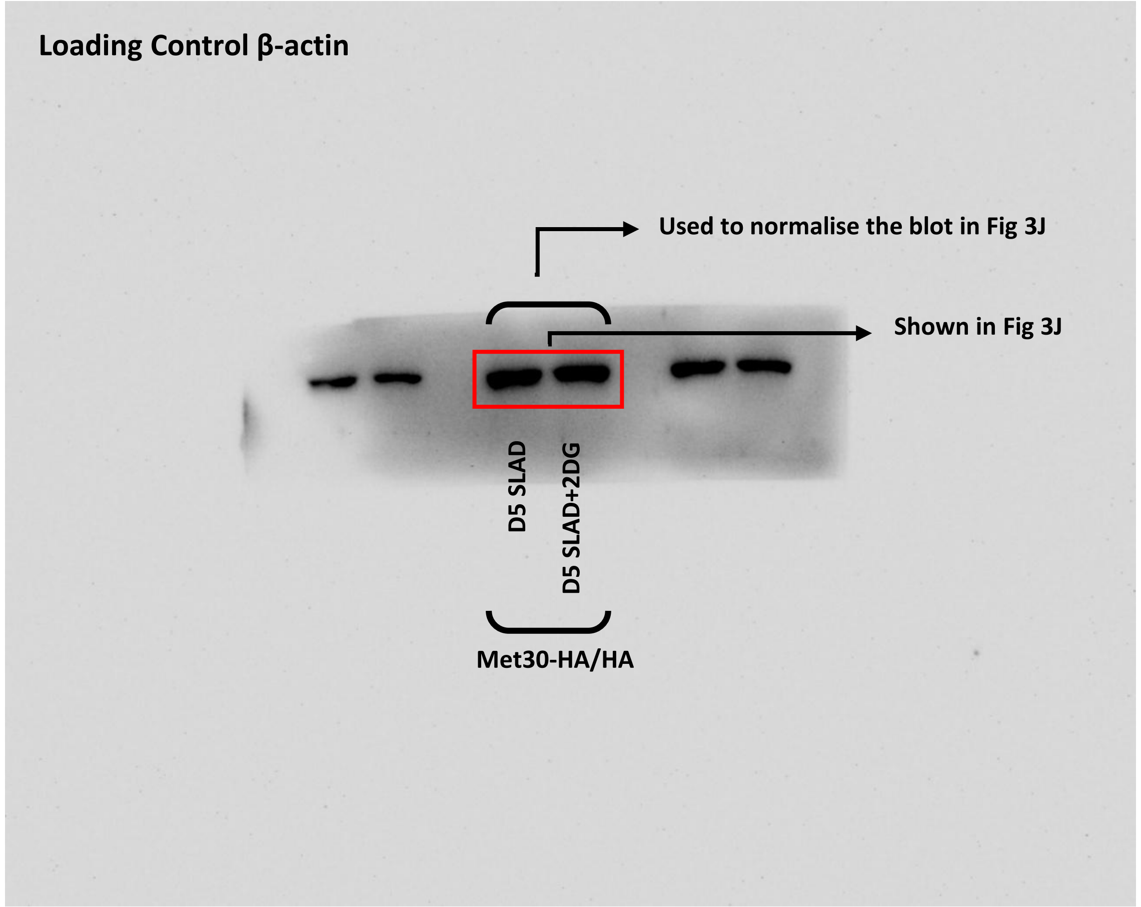

Supplement: Figure 3—source data 1. [file elife-109075-fig3-data1.zip › Figure 3-source Data 1/Figure 3-source data, uncropped and labelled blots.pdf]
