# Supplementary material for: Glycolysis-dependent sulfur metabolism orchestrates morphological plasticity and virulence in fungi
Source: eLife. 2026 Feb 6;14:RP109075. doi: 10.7554/eLife.109075 (PMC12880806; doi:10.7554/eLife.109075)
Supplement: Supplementary file 1. [file elife-109075-supp1.docx]

**Supplementary file 1. Yeast Strains Used in this Study**

| **Strain** | **Genotype** |
| --- | --- |
| ***S. cerevisiae* ∑1278b** | |
| Wild-type | *MATa/α* |
| SV18 | *∆adh1::G418/∆adh1::HygB MATa/α* |
| SV21 | *∆pfk1::HygB/∆pfk1::G418 MATa/α* |
| SV220 | *∆gpa2::NAT/∆gpa2::G418 MATa/α* |
| SV270 | *∆met32::NAT/∆met32::G418 MATa/α* |
| SV320 | *MET32-6xHA::G418/MET32-6xHA::NAT MATa/α* |
| SV394 | *CYS3-6xHA::NAT/CYS3-6xHA::G418 MATa/α* |
| SV395 | *CYS4-6xHA::NAT/CYS4-6xHA::G418 MATa/α* |
| SV402 | *MET10-6xHA::NAT/MET10-6xHA::G418 MATa/α* |
| SV404 | *MET16-6xHA::NAT/MET16-6xHA::G418 MATa/α* |
| SV431 | *∆met30::HygB MATa/α* |
| SV451 | *MET30-6xHA::NAT/MET30-6xHA::G418 MATa/α* |
| SV461 | *MET4-6xHA::NAT/MET4-6xHA::G418 MATa/α* |
| SV463 | *∆met30::HygB/MET4-6xHA::NAT/MET4-6xHA::G418 MATa/α* |
| SV473 | *MET16-6xHA::NAT/MET16-6xHA::G418/∆met30::HygB MATa/α* |
| SV475 | *MET32-6xHA::G418/MET32-6xHA::NAT/∆met30::HygB MATa/α* |
| SV476 | *CYS3-6xHA::NAT/CYS3-6xHA::G418/∆met30::HygB MATa/α* |
| ***S. cerevisiae* CEN.PK** | |
| Wild-type | *MATa/α* |
| SV109 | *∆adh1::G418/∆adh1::NAT MATa/α* |
| SV117 | *∆pfk1::NAT/∆pfk1::G418 MATa/α* |
| ***C. albicans* SC5314** | |
| Wild-type | *MATa/α* |
| SV308 | *∆pfk1::FRT/∆pfk1::SAT-FLP MATa/α* |
