# Supplementary material for: Glycolysis-dependent sulfur metabolism orchestrates morphological plasticity and virulence in fungi
Source: eLife. 2026 Feb 6;14:RP109075. doi: 10.7554/eLife.109075 (PMC12880806; doi:10.7554/eLife.109075)
Supplement: Supplementary file 2. [file elife-109075-supp2.docx]

**Supplementary file 2. Oligonucleotides Used in this Study**

| Oligonucleotides | Sequence (5’ to 3’) |
| --- | --- |
| **Primers for *S. cerevisiae* Gene Editing and Genotyping** | |
| ADH1 KO F1 | AGCTATACCAAGCATACAATCAACTATCTCATATACAATGCGGATCCCCGGGTTAATTAA |
| ADH1 R1 | ATTTAATAATAAAAATCATAAATCATAAGAAATTCGCTTAGAATTCGAGCTCGTTTAAAC |
| ADH1 KO Chk | CGTTGTTGTCTCACCATATC |
| PFK1 KO F1 | TTTTATATAAAAAATCTGAAACAAAATCATATCAAAGATGCGGATCCCCGGGTTAATTAA |
| PFK1 R1 | CTCCTTTTGCTTAACTTAAACTTTTCATTGCAATCATTCAGAATTCGAGCTCGTTTAAAC |
| PFK1 KO Chk | AGGATGAGAAAGTGAAATCG |
| GPA2 KO F1 | TTACAGCACAAATCACGCGTATTTTCAAGCAAATATCATGCGGATCCCCGGGTTAATTAA |
| GPA2 R1 | AGAGGCATGCAGTTTTGTCTCTGTTTTAGCTGTGCATTCAGAATTCGAGCTCGTTTAAAC |
| GPA2 KO Chk | ACATGACTACTGCCTCGTTC |
| MET32 KO F1 | AAGTCAAGAGGTATTATAAATTTCAAAAAAGTACCAAATGCGGATCCCCGGGTTAATTAA |
| MET32 R1 | ATTTTCTTATTTGAGAAGATACACGCTATTTACTCTTTCAGAATTCGAGCTCGTTTAAAC |
| MET32 KO Chk | GATGGTTTTTCGTCCCTTAC |
| MET32 C tag F1 | TCATCGTCAAGATAACAACCACAATGGTAGCAGTAATGGCCGGATCCCCGGGTTAATTAA |
| MET32 C tag Chk | AAAAAGGAAAACGCATTACC |
| CYS3 C tag F1 | TTTGGAAGACATCAAGCAAGCCTTGAAACAAGCCACCAACCGGATCCCCGGGTTAATTAA |
| CYS3 R1 | AAAGGTCCGGTCGAAGGCAGAGACGTGGCACTGGCGATTAGAATTCGAGCTCGTTTAAAC |
| CYS3 C tag Chk | CAGAGGTTTGAAGACTTTGC |
| MET10 Ctag F1 | AGAATTAAAGGAAGCATCAAGATACATTTTAGAAGTCTACCGGATCCCCGGGTTAATTAA |
| MET10 R1 | CAATAAATAGATATTTAGTTTTTATTACTATATTAATTTAGAATTCGAGCTCGTTTAAAC |
| MET10 Ctag Chk | TGGTTTAGGTACTGGTTTGG |
| MET16 Ctag F1 | TGAAGCCAGCCGATTCGCGCAATTTTTAAAGCAAGATGCCCGGATCCCCGGGTTAATTAA |
| MET16 R1 | GTCACATACATATGGTTATATATCGTACTCTATCTATCTAGAATTCGAGCTCGTTTAAAC |
| MET16 Ctag Chk | AATACGGGGATTTCTTATGG |
| MET4 Ctag F1 | AAGCTTAAAGAAGCAAATTTTTGAGAAGGTTCAGAAAGAACGGATCCCCGGGTTAATTAA |
| MET4 R1 | TATATATATATATAATTAAACTGTATAGTCTGTTATTTTAGAATTCGAGCTCGTTTAAAC |
| MET4 Ctag Chk | GAGCCAACTTTAAATGCAAG |
| CYS4 Ctag F1 | CCATATCGTTACTAAGATGGATTTACTGAGCTACTTAGCACGGATCCCCGGGTTAATTAA |
| CYS4 R1 | TTCTATGTTTGCTTTTATTTGAAGCGTGGGTTCTTATTTAGAATTCGAGCTCGTTTAAAC |
| CYS4 Ctag Chk | TTAAGGAAACCGCTAAGGTC |
| MET30 KO F1 | AGGGGTGTGTGTTTGGTGATTTATAAAGGAGAAGGGCATGCGGATCCCCGGGTTAATTAA |
| MET30 R1 | CGGATGTTTTTGACCAAGAAAAGACCACACACAGGTCCTAGAATTCGAGCTCGTTTAAAC |
| MET30 KO Chk | AGAGATAACTGCAGGGTGTG |
| MET30 Ctag F1 | ATTTGGGTGCGTAAAAATGTACAAATTCGATCTCAATGATCGGATCCCCGGGTTAATTAA |
| MET30 Ctag Chk | AGGATGACCCAACAATGAC |
| **Primers for *C. albicans* Gene Editing and Genotyping** | |
| Psfs2a PFK1 F1 | ATTTGAATAGCTTTTTCTTATTATTGAATTCTTTAATTTATCGTTTATTGCAAACAATCAATTATTCCTTATTATTTTGGGCAGATAACGAATTAGAAATGAAGTTCCTATACTTTCTAG |
| Psfs2a PFK1 R1 | CAACACATTGAATTACATATATGAGTATAAAAAATCAAACTATACTGGGGACTACAAATCTTACTATTTTACACAACTCCTTATCTTCTGTTCTTCATTTGAAGTTCCTATTCTCTAGAA |
| Psfs2a SAT-FLP F1 | AACATTGGATGCTGAGAACC |
| Psfs2a SAT-FLP R1 | GCACATAATGCTATTTTCTC |
| PFK1 ApaI UF1 | GAGAGGGCCCGTGATGCCTAACTTCTTGGT |
| PFK1 SacII DR1 | CTCTCCGCGGTAGGCTGAATTGCTCGTATG |
| PFK1 KO R1 Chk | GTACCAATTGAAGTACCACC |
| FRT F1 | CTTTCTAGAGAATAGGAACT |
| FRT R1 | AGTTCCTATTCTCTAGAAAG |
| **Primers for RT-qPCR** | |
| *S. cerevisiae* ACT1 F1 | CGTCTGGATTGGTGGTTCTATC |
| *S. cerevisiae* ACT1 R1 | GGACCACTTTCGTCGTATTCTT |
| *S. cerevisiae* MET32 F1 | TCCTCAATGTGGCAAAGGT |
| *S. cerevisiae* MET32 R1 | CACCACCCGCAGTTAGTAAT |
| *S. cerevisiae* MET3 F1 | CAGAGTTGAGACGCCGTTTA |
| *S. cerevisiae* MET3 R1 | GTCTTGGTGGGTTGGATTCT |
| *S. cerevisiae* MET5 F1 | CATCGCCGTTCCTCCATATAAC |
| *S. cerevisiae* MET5 R1 | CCCATACCACCACCAACAAA |
| *S. cerevisiae* MET17 F1 | CCCTGGTTTAGCATCTCATTCT |
| *S. cerevisiae* MET17 R1 | CAGTTTCCTTGTCGGCATTTG |
| *C. albicans* ACT1 F1 | TTGGATTCTGGTGATGGTGTTA |
| *C. albicans* ACT1 R1 | TCAAGTCTCTACCAGCCAAATC |
| *C. albicans* MET32 F1 | CACCAACACAACACCATCAAC |
| *C. albicans* MET32 R1 | TCCAATTCCACCACCAGTAATC |
| *C. albicans* MET3 F1 | GTTCCAACAACAAACCCAACTC |
| *C. albicans* MET3 R1 | TCACCATCAGCATTACCAGAAG |
| *C. albicans* MET5 F1 | TTTATGGTGCGTGTTCGTTTAC |
| *C. albicans* MET5 R1 | TAGCCCTGGTGGTCAATTTC |
| *C. albicans* MET10 F1 | GGGTTGATCCAAAGGGAAGAT |
| *C. albicans* MET10 R1 | GACAATGGTGGCAACTTCATAAC |
| *C. albicans* MET17 F1 | TCAAGGACATCACCAACACC |
| *C. albicans* MET17 R1 | CTTAGAGTCACCCACATTAGCC |
| *C. albicans* ALS3 F1 | GGTGGCACTGATTCGGTTAT |
| *C. albicans* ALS3 R1 | GCGGTAATGGTAGTGGTAGTTG |
| *C. albicans* ECE1 F1 | TTGGCAACATTCCACAAGTAATC |
| *C. albicans* ECE1 R1 | CAGCAATGATACCAGCAACAAC |
| *C. albicans* HWP1 F1 | CTCTACGACTGAAGGTGCTATTC |
| *C. albicans* HWP1 R1 | GTAGAAATAGGAGCGACACTTGA |
| *C. albicans* HYR1 F1 | CACATCAAGTCCTGGTCAATCTA |
| *C. albicans* HYR1 R1 | TGGAACAGTGGTGAAGATAGTG |
| *C. albicans* IHD1 F1 | GGCTCTCAAGGTCAATCTACAA |
| *C. albicans* IHD1 R1 | ACTAGCACCATCGTTACCATTAG |
| *C. albicans* RBT1 F1 | CCATCTGCTAACTCCTCATACAC |
| *C. albicans* RBT1 R1 | CAAGAATGCAGCAAGACCAATAA |
| *C. albicans* SAP6 F1 | GAGTGTTCTTGCTTTCGCTTTAT |
| *C. albicans* SAP6 R1 | CATCTGGATCAACAAGGGATCT |
